# Attributes of Drying Define the Structure and Functioning of Microbial Communities in Temperate Riverbed Sediment

**DOI:** 10.3389/fmicb.2021.676615

**Published:** 2021-06-14

**Authors:** José Schreckinger, Michael Mutz, Clara Mendoza-Lera, Aline Frossard

**Affiliations:** ^1^Department of Freshwater Conservation, Brandenburg University of Technology Cottbus-Senftenberg, Bad Saarow, Germany; ^2^Institute of Environmental Sciences, Koblenz-Landau University, Landau, Germany; ^3^Swiss Federal Institute for Forest, Snow, and Landscape Research (WSL), Birmensdorf, Switzerland

**Keywords:** bacteria, community respiration, dissolved organic matter, dry riverbed, extracellular enzyme activities, fungi, microbial function, sediment

## Abstract

Combined effects of climate change and increasing anthropogenic water demand have increased and extended dry period occurrences in rivers worldwide. Riverbed drying can significantly affect sediment microorganisms, crucial drivers of biogeochemical processes in lotic systems. In this study, we evaluated how sediment bacterial and fungal community structure and composition (based on 16S rRNA gene and ITS metabarcoding) and microbial functions (community respiration and extracellular enzymatic activities) respond to different riverbed drying intensities over 90 days. Riverbed sediment collected in a flowing reach of the Spree river in northeastern Germany was dried under different rates in outdoor mesocosms during the summer months of 2018. Our results demonstrate that drying attributes (duration and intensity) and sediment organic matter (OM) content play a crucial role in sediment microbial community assembly and functioning throughout drying. Milder drying surprisingly triggered a more rapid and drastic change in the microbial community composition and diversity. After 90 days of drying, Bacilli (Firmicutes) became the dominant bacterial class in most treatments, except in sediments with low OM content under the most severe drying treatment. Fungal amplicon sequence variants (ASVs) from Dothideomycetes (Ascomycota) had by far the highest relative abundance in all our treatments at the end of the drying experiment, making up 65.1% to 94.0% of the fungal reads. CO_2_ fluxes, a proxy for sediment community respiration, were rapidly and strongly affected by drying in all treatments. Our results imply that even short riverbed drying periods are likely to have significant consequences for the biogeochemical dynamics in recently formed non-perennial temperate rivers.

## Introduction

Worldwide, about half of the length of the global fluvial network dries at some point in space and time (Datry et al., 2014). Such reaches are called intermittent rivers and ephemeral streams (IRES). While IRES naturally occur mostly in arid and semi-arid climates, water demand, and climate change are currently increasing the number and extent of IRES globally (Palmer et al., 2008; Steward et al., 2012; Pekel et al., 2016). Prolonged summer droughts, such as the one in Central Europe in 2018 (Toreti et al., 2019; Vogel et al., 2019), are likely to promote the transition of several temperate river systems from perennial to intermittent flow regimes. Although research on IRES has increased, their biogeochemical processes have not been as broadly studied as they have been for perennial reaches (Steward et al., 2012; Bernal et al., 2013). Moreover, understanding the biogeochemical implications of riverbed drying requires knowledge of the influence of this process on microbial community structure and function (von Schiller et al., 2017), and environmental attributes need to be considered for a more comprehensive understanding.

Dry riverbed sediments can functionally and structurally be considered as early-stage soils (Arce et al., 2019). As in soils, rainfall, temperature, and evaporation play an essential role in sediment moisture content in dry riverbeds, and they are crucial drivers of biogeochemical processes such as organic matter (OM) decomposition and mineralization (Larned et al., 2010; Steward et al., 2012). Riparian vegetation cover may reduce drying intensity by providing shadow, which reduces sediment temperature and evaporation while retarding the loss of riverbed moisture. Riparian vegetation cover may also prevent microbial community exposure to harmful solar radiation, such as Ultraviolet (Romaní et al., 2017).

Temporary drying of river channels can significantly compromise the function and structure of the riverbed microbial community (Junk et al., 1989; Humphries and Baldwin, 2003; Datry et al., 2014), which is responsible for a large proportion of the carbon and nutrient processing in river systems (Cole et al., 1988; Duarte and Prairie, 2005). Dry riverbeds are, in general, sources of CO_2_ fluxes to the atmosphere (Keller et al., 2020). However, the duration of non-flow periods and the intensity of drying are known factors influencing microbial community assembly and C-transformation in riverbeds (Marxsen et al., 2010; Gionchetta et al., 2020). The frequency and amount of rainfall events on dry riverbeds may also trigger changes in dry sediment microbial structure and activity (Arce et al., 2018).

The bacteria and fungi living in riverbed sediment play a crucial role in riverbed biogeochemical processes. During riverbed drying, however, the activity and abundance of bacterial communities tend to decline (Tzoraki et al., 2007; Amalfitano et al., 2008). Changes in microbial community structure and functions subsequent to riverbed drying may be attributed to increasing UV radiation (Bergeron and Vincent, 1997), to reduced advection and diffusion of C and nutrients (Zoppini et al., 2010; Manzoni et al., 2017), and to changes in sediment physical structure, as observed in soils (Borken and Matzner, 2009). The impact of riverbed drying on the bacterial community structure remains, however, unclear; while only minor changes in the composition of sediment bacterial communities were found after drying events in some studies (Marxsen et al., 2010; Timoner et al., 2014), substantial changes were reported in others (Pohlon et al., 2013; Gionchetta et al., 2019).

Bacterial and fungal cells resist drought events by entering dormancy and by reducing their function and growth (Cook and Orchard, 2008; Moyano et al., 2013; Sabater et al., 2016). Sediment fungal communities tend to cope with riverbed drying better than bacteria (Gionchetta et al., 2018), due to fungal traits of spore mobility and mycelial growth, which help them to search for nutrients and water through air-filled sediment pores (De Boer et al., 2005). Fungi additionally have thicker hydrophobic cell walls, which are more efficient in reaching osmotic equilibrium (Baschien et al., 2013; Manzoni et al., 2017). While riverbed drying has been reported to negatively affect fungal biomass in leaf packs accumulated on the riverbed (Bruder et al., 2011; Foulquier et al., 2015), only a few studies have involved assessments of changes in fungal diversity in drying sediments (Gionchetta et al., 2020).

Drying also affects microbial metabolism and activities in river sediments, often reducing rates of OM decomposition and mineralization (Larned et al., 2010; Romaní et al., 2013). Moreover, the chemical composition of dissolved organic matter (DOM), the primary C and nutrient source for heterotrophic microbial metabolism in lotic environments (Tank et al., 2010), is known to be altered subsequent to riverbed drying (del Campo et al., 2019), contributing to large changes in the structure and function of sediment microbial communities (Obermann et al., 2009; Freixa et al., 2016).

In this study, we aimed to assess the implications of riverbed drying on microbial communities and OM mineralization. We used outdoor mesocosms to assess the effect of drying intensity on sediment-associated bacterial and fungal community structure and functions, as well as on leached DOM, throughout 90 days of drying. We hypothesized that (1) higher drying intensity would result in a more rapid change in sediment microbial community structure and functions. Moreover, we expected (2) microbial functions, particularly CO_2_ production, to be more rapidly affected by drying than bacterial and fungal community structures. Finally, we expected (3) the riverbed sediment OM concentration and DOM quantity and quality to be significant factors impacting microbial community composition and activities during the drying process.

## Materials and Methods

### Experimental Design

The experimental set-up consisted of 54 outdoor mesocosms (2.4 L cylindrical polypropylene pots with four drainage holes in the bottom) filled with a 5-cm-thick gravel layer (commercial “Flairstone” gravel, 8–16 mm; Hornbach, Biel, Switzerland), providing support and drainage for top 10 cm sediment sample layer (2.2 L). Sandy loose sediment was collected in June 2018 from the top 10 cm of fully submerged riverbed close to an open shore of the Spree river (near Cottbus, Germany, 51° 50’08.1” N 14° 20’42.5” E, elevation 70 m above sea level). The collected sediment was homogenized and directly filled into 27 of the mesocosms. The other 27 mesocosms were each filled with a homogenized mix containing 1.76 L of sediment enriched with 0.44 L of fine organic matter (OM, particles < 2 mm) collected from a lentic part of the river next to the sediment sampling site. The addition of OM increased the OM content in enriched sediment (i.e. sediment with OM addition; 5.82 ± 0.5 mg AFDW g DW^–1^; AFDW = ash-free dry weight, DW = dry weight) by 75% compared to non-enriched sediment (i.e. sediment without OM addition; 3.32 ± 0.3 mg AFDW g DW^–1^). The sediment layer in the microcosms was 10 cm thick and had a diameter of 14 cm. Subsequent to filling, mesocosms were transported to the research station’s outdoor facility at Bad Saarow and buried in sand so that the tops were flush with the ground surface, simulating a natural dry shore environment. The two sediment types (non-enriched and enriched) were exposed for 10, 30, or 90 days (June to September 2018) to one of three drying intensities: (i) high: no rain and no shade, (ii) moderate: with rain but no shade, and (iii) low: with rain and shade. In order to control rain events, all the mesocosms were placed under a roof (transparent PVC). Rainfall simulations were applied for moderate and low treatments every week. The rainfall simulations consisted of adding, drop-wise, 22 mL of artificial rainwater, equivalent to the mean precipitation amount in the experimental period (Chicken Creek catchment weather station of the Brandenburg University of Technology [BTU] Cottbus). The artificial rainwater consisted of ultrapure water with 0.052 mmol L^–1^ Cl 0.03 mmol L^–1^ Ca, 0.08 mmol L^–1^ SO4, 0.06 mmol L^–1^ Na, and 0.02 mmol L^–1^ K (Zönnchen et al., 2014). The transparent PVC roof on top of all the mesocosms reduced incoming light by 25% (HOBO UA-002-08 Pendant data loggers, ONSET, Bourne, MA, United States). Shade equivalent to a dense vegetation cover was simulated by placing cardboard structures about 10 cm above the low-drying-intensity mesocosms, reducing sunlight by 94% relative to the other mesocosms under the PVC roof. Sediment moisture and temperature were recorded every 30 min with probes (5TE sensors with Em50 ECH2O dataloggers, METER Group, Pullman, WA, United States) placed at 3 cm and 6 cm depth in nine extra mesocosms filled with non-enriched sediment, three of each drying intensity (high, moderate and low).

At each sampling date during drying (after 10, 30, and 90 days), we destructively sampled 18 mesocosms (3 from each treatment and sediment type). The sediment was homogenized, subsampled and immediately processed for sediment physicochemical parameters, DOC quantity and DOM quality. For extracellular enzymatic activity (EEA) and microbial community structure, subsamples were stored at −20°C until processing.

### Sediment Physiochemical Characteristics

Sediment dry weight (DW) and gravimetric water content (GWC) were measured after drying samples in an oven at 105°C overnight. Organic matter content (AFDW) was estimated by weight loss after ignition (muffle oven at 500°C for 3 h) of dry samples. Sediment grain-size distribution was determined by weighing the ignited sediment residues that passed through different sieve mesh sizes (2, 0.63, 0.2, and 0.063 mm) upon mechanical shaking (10 min). The pH of the sediment was determined prior to the drying experiment, under flowing conditions, by mixing 10 g of sediment with 20 ml of ultrapure water (1:2) and stirring for 30 min. The mix was then allowed to stand for 1 h and measurements were recorded using a pH meter 540 GLP (WTW, Weilheim, Germany).

### Bacterial and Fungal Amplicon Sequencing

We used DNeasy Powersoil kits (Qiagen, Hilden, Germany) to extract sediment total DNA. Extracted DNA concentrations were quantified with the PicoGreen dsDNA quantification kit (Thermo Fisher Scientific, Waltham, MA, United States). We targeted and amplified the 16SrRNA*_V3–V4_* genomic region (for bacteria) using primer pairs 341F/806R and the ITS2 region (for fungi) with the primer pairs ITS3/ITS4 (Perez-Mon et al., 2020). The PCR reaction mix for bacterial and fungal amplifications consisted of: GoTaq^®^ Flexi Buffer 5x (5 μL), MgCl_2_ 25 mM (2.5 μL), forward and reverse primer (each 0.2 μL), dNTP 10mM (0.5 μL), BSA (0.5 μL), Hot start Taq (0.25 μL), dd H_2_0 (8.4 μL), and DNA extract (10 ng). PCR amplification steps for 16s rRNA started with 2 min at 95°C, followed by 36 amplification cycles, which included a denaturation step at 94°C for 40 s, primer annealing at 58°C for 40 s, elongation at 72°C for 1 min and a final step at 72°C for 10 min. The same amplification steps were followed for the ITS PCR with 38 amplification cycles. Each PCR was run in triplicate and their product pooled. Bacterial and fungal amplicons were sent to the Génome Québec Innovation Centre at McGill University (Montreal, Canada) where they were purified, quantified, barcoded and paired-end sequenced using the Illumina MiSeq v3 platform (Illumina Inc., San Diego, CA, United States). Through the analysis, we included one negative control from DNA extraction to sequencing.

### Sequence Analysis

For quality control, filtering and merging of bacterial and fungal reads we used dada2 pipeline to inference amplicon sequencing variants (ASVs) (Callahan et al., 2016), using R version 4.0.5. We trimmed and truncated sequences using filterAndTrim function, discarding truncated reads with more than 2 expected errors (maxEE) or first instance quality scoreless or equal to 2. PCR primers were removed previously to merging pairs using trim Left function. After merging, we accepted read lengths > 200 bp for ITS and >300 bp for 16S reads. Chimeras were removed using remove Bimera Denovo function. ASVs were assigned taxonomically using DECIPHER package function IdTaxa (Murali et al., 2018), defining processor as “NULL.” Then, 16S reads were queried against the SILVA v.138.1 (McLaren et al., 2021) reference database (bacteria), and ITS reads against the UNITE v.8.2 (Abarenkov et al., 2020) reference database (fungi). A total of 544,555 bacterial reads (6,910 ASVs) and 56,096 fungal reads (2,589 ASVs) were recovered. We removed all ASVs that were not assigned to the bacterial lineage, including ASVs with unclassified domains. Besides, we removed ASVs that were taxonomically assigned as chloroplast and mitochondria.

### Bacterial and Fungal Abundance

The abundance of bacterial 16S rRNA gene and fungal ITS region copies was assessed using quantitative real-time PCR (qPCR) on an ABI7500 Fast Real-Time PCR system (Applied Biosystems, Foster City, CA, United States). The same primers (without the barcodes) and cycling conditions employed for the amplicon sequencing were used for 16S rRNA and ITS qPCR. We used three standard curves for each targeted region, obtained using tenfold serial dilution (10^–1^ to 10^–9^ copies) of plasmid generated from cloned targets.

### CO_2_ Fluxes and Community Respiration (CR)

Throughout the experiment, over 90 days of drying, weekly CO_2_ fluxes (a proxy for community respiration) were measured in a subset of 18 mesocosms (*n* = 3 per treatment) by tightly placing a closed acrylic glass chamber with a headspace over each mesocosm. CO_2_ concentrations (ppm) in the chamber were recorded with a K33 LP T module (Senseair AB, Delsbo, Sweden) under dark conditions. CO_2_ fluxes (ppm) were converted to μgCO_2_ g DW^–1^ h^–1^ and the decline in CO_2_ production with ongoing drying was estimated by fitting the following exponential function in Origin Pro software (OriginLab Corporation, Northampton, MA, United States):

$$y=y_{o}\,A\,e^{-x/t}$$

Where y_o_ is the offset, A the amplitude or scaling factor, and t the time of the exponential decay model. Exponential decay rates k are equal to t^–1^.

Before the drying experiment, sediment community respiration (CR) was measured under flowing conditions as dissolved oxygen (DO) consumption in 30 ml glass columns (FORTUNA OPTIMA, Poulten & Graf, GmbH, Wertheim, Germany). We perfused sediment with artificial river water (Lehman, 1980; Zlatanović et al., 2017) enriched with 200 μg L^–1^ NH_4_-N and 10 μg L^–1^ PO_4_^3–^, mimicking concentrations in Spree river water (Landesamt für Umwelt, Cottbus Sandower Brücke, 2015). The artificial stream water was pumped through the columns filled with non-enriched or enriched Spree riverbed sediment (*n* = 3) at a rate of 0.04 ml min^–1^ to generate a gentle interstitial flow, similar to that observed in shallow, sandy riverbed sediments in the region (Angermann et al., 2012). Columns containing sediment were placed vertically inside a climate chamber under dark conditions at a constant temperature of 17°C (mean Spree water temperature in late June, Landesamt für Umwelt Brandenburg, Cottbus Sandower Brücke, 2015). After 12 h of water perfusion, we recorded the DO concentration at the column’s in-flow and out-flow (oxygen microoptodes, PM-PSt7, Pre Sens, Germany) and subtracted the DO loss measured in a column filled with water only (blank). For comparative purposes, CR under flowing conditions was converted from DO consumption to CO_2_ production considering a respiratory coefficient of 0.85 (Bott, 1996). DO and CO_2_ measurements were additionally standardized to 20°C using a Q_10_ temperature coefficient of 2 (Winkler et al., 1996).

### Extracellular Enzymatic Activities (EEAs)

Extracellular enzyme assays were conducted to determine the potential activity of β-glucosidase, β-xylosidase, alkaline phosphatase, chitinase, leucine aminopeptidase, phenol oxidase, and phenol peroxidase (Frossard et al., 2012). For the assays, we added 2 g of sediment sample and 60 mL of buffer solution (Trishydroxyaminomethane 0.1 M) into an autoclaved glass beaker. After stirring the sediment slurry for 30 s, 200 μL was pipetted and transferred four times into a microplate well (four technical replicates), along with 50 μL of 200 μM substrate analog solution. We used substrate analogs attached to fluorescent 4-methylumbelliferone (MUB) for β-glucosidase, β-xylosidase, alkaline phosphatase and chitinase, and to 7-amino-4methylcoumarin (AMC) for leucine aminopeptidase. Phenol oxidase and phenol peroxidase were measured through the oxidative reaction of the substrate analog dihydroxyphenylalanine (L-DOPA). For PP, 10 μL of 0.3% H_2_O_2_ was added in addition to the substrate analog. We incubated filled microplates for 1.5 h at a constant temperature (17°C) in the dark and with constant mild stirring. Fluorescence and absorbance measures were read with an Infinite 200 fluorometer (Tecan, Männedorf, Switzerland) with excitation/emission wavelengths of 365/445 nm for β-glucosidase, β-xylosidase, alkaline phosphatase and chitinase and 365/450 for leucine aminopeptidase, and absorbance was assessed at 460 nm for phenol oxidase and phenol peroxidase. Control wells were included in the assays to correct variations in the fluorescence and absorbance background of the sample slurries and the substrate analog. Quenching was also assessed and corrected by calculating the quotient of fluorescence of each sample spiked with a fluorescent standard (MUB or AMC) and the same standard with buffer only. Potential enzyme activity was calculated in μmol h^–1^ mL^–1^ and standardized to 20°C using a Q_10_ temperature coefficient of 2.

### DOM Quality and Quantity

Aqueous DOM leachates were prepared to assess the DOM quality and quantity by placing 8 g sediment in previously acid-washed 50 ml falcon tubes containing 40 ml of NaCl solution (220 mg L^–1^). The falcon tubes were kept at 4°C in dark conditions for 24 h. Subsequently, they were centrifugated for 10 min at 5000 rpm. Leachates were filtered at 45 μm into a precombusted glass vial. We used a TOC analyzer (TOC–Vcph, Shimadzu, Kyoto, Japan) for the quantification of dissolved organic carbon (DOC). The DOM of the filtered leachates was spectroscopically characterized using an Aqualog Analyzer (Horiba Scientific, Kyoto, Japan). We ran excitation-emission matrices (EEMs) and absorbance measures simultaneously. Emissions were recorded at 210 to 618 nm with 1.59 nm increments and fluorescence intensities were recorded at 230 to 600 nm with 5 nm increments. Blanks (Milli-Q water) and inner filter effects were corrected with Aqualog software. The humification index (HIX), biological index (BIX), fluorescence index (FI) and SUVA254 index (Zsolnay et al., 1999; Weishaar et al., 2003; Jaffé et al., 2008; Huguet et al., 2009) were calculated with the “staRdom” package (version 1.0.18) in R (R Core Team, 2020).

### Data and Statistical Analysis

All statistical tests were performed and all figures were created in R version 4.0.5. We defined a statistical significance level of 0.05. The difference between sediment types (non-enriched and enriched) was tested under flowing conditions (prior to drying) using one-way probabilistic permutation multivariate analysis (PERMANOVA). During the drying phase, we used three-way PERMANOVAs to test differences between sediment types (non-enriched and enriched), drying intensity treatments (low, moderate and high), and drying durations (10, 30, and 90 days). Additionally, we used a two-way PERMANOVA to test differences in community respiration exponential decay rates between sediment types and drying intensity treatments. The probabilistic permutation test does not require any data assumptions. We set the tests for sequential SS and 10^–6^ stop interactions, using the *aovp* function in the “ImPerm” R package. We then ran the *TukeyHSD* function (“stats” package version 3.6.2) as a post-hoc test to determine significant differences between individual factor levels.

Bacterial and fungal richness and the Shannon index were calculated at the ASV-level using phyloseq package after performing rarefaction to the lowest sample read count. We ran a non-metric multidimensional scaling (NMDS) PERMANOVA (*adonis* function, “vegan” package) to assess the β-diversity community structure and pairwise post-hoc tests to assess differences among sediment types, drying intensities and drying durations at the ASV-level. For each drying treatment, after 10, 30, or 90 days of drying, we calculated the log2-fold change response of ASV’s count from each bacterial and fungal at class-level relative to the corresponding before-drying ASV’s assembly, using the “Deseq” package (Brantschen et al., 2020). Positive significant log2-fold change responses were defined as tolerant ASVs and negative change responses were defined as sensitive. We discarded ASV’s with fewer than 10 sequences for the previously mentioned analysis and assembled a heatmap consisting of log2-fold change values. Finally, we generated a correlation matrix with sediment properties using the *cor* function in the “stats” package. All figures were made using the “ggplot2” and “vegan” packages.

## Results

### Physicochemical Parameters

Prior to drying, under flowing conditions, the pH in enriched sediment (7.91 ± 0.01) was slightly, though not significantly, lower than in non-enriched sediment (8.01 ± 0.03). The enriched and non-enriched sediments had similar grain size distributions (Supplementary Table 1). Throughout drying, the average hourly temperatures in non-enriched sediment ranged from 7.7 to 33.9°C for the low-intensity drying treatment, from 8.2 to 39.8°C for the moderate treatment, and 8.2 to 42°C for the high-intensity treatment (Supplementary Figure 1). The overall average sediment temperature for the low-intensity treatment (21.4 ± 0.2°C) was significantly lower than for the moderate (22.8 ± 0.2°C) and high-intensity (23.2 ± 0.2°C) treatments (Supplementary Table 2). Volumetric water content (VWC) decreased exponentially during drying in all three treatments (Supplementary Figure 1). During the early drying phase (between 0 and 30 days), we observed that sediment under the low-intensity drying treatment retained a higher VWC content than sediment exposed to the moderate- and high-intensity treatments. Unfortunately, due to one sensor’s failure, we could not run statistical tests for the VWC data. However, gravimetric water content (GWC) decreased significantly in all treatments with increasing drying time. For both sediment types, the low-intensity drying treatment resulted in a higher gravimetric water content (GWC) than the moderate- and high-intensity drying treatments after 10 and 30 days, but not anymore after 90 days (Supplementary Figure 2 and Supplementary Table 2). The moderate- and high-intensity drying treatments did not differ significantly in GWC in either sediment type.

### Bacterial and Fungal Abundance

**FIGURE 1:**
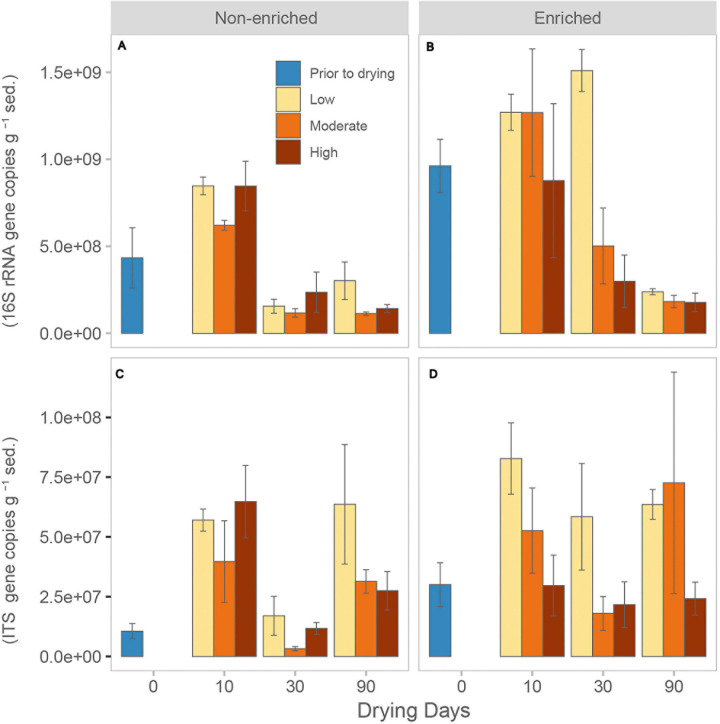
Average (mean + SE, *n* = 3) number of 16S rRNA gene copies for **(A)** non-enriched and **(B)** enriched sediment, and average number of ITS gene copies for **(C)** non-enriched and **(D)** enriched sediment under each drying intensity (low, moderate, and high) at different drying durations (0, 10, 30, and 90 days).

Prior to drying, enriched sediment had a significantly higher bacterial abundance, averaging 9.6 × 10^8^ ± 2.6 × 10^8^ gene copies, compared with non-enriched sediment, with only 4.3 × 10^9^ ± 3 × 10^8^ gene copies (Table 1 and Figure 1). After 30 days of drying, bacterial abundance decreased considerably in all treatments, except for the low-intensity drying treatment in enriched sediment. Nevertheless, after 90 days of drying, the bacterial abundance was similarly low under all treatments (average over all treatments 1.9 × 10^8^ ± 1 × 10^8^; Figure 1). Under flowing conditions (prior to drying), fungal abundance was also significantly greater in enriched sediments (3.00 × 10^7^ ± 1.59 × 10^7^ gene copies) than in non-enriched sediments (1.06 × 10^7^ ± 5.50 × 10^6^ gene copies). Fungal abundance was significantly higher in the low-intensity drying treatment than in the high-intensity treatment (Table 1 and Figure 1). Interestingly, fungal abundance in most treatments significantly decreased between days 10 and 30 but then increased again at 90 days of drying.

**TABLE 1:** *P*-values from PERMANOVAs and post-hoc tests (Tukey’s HSD and pairwise) related to microbial communities diversity and abundance.

		**Gene abundance**		**Richness**		**Shannon diversity**		**β-Diversity**	
		**Prior to drying**	**Drying**	**Prior to drying**	**Drying**	**Prior to drying**	**Drying**	**Prior to drying**	**Drying**
**Bacterial**									
	Sediment type	**0.001**	** <0.001**	0.201	1.000	1.000	0.7068	0.100	** < 0.001**
	Drying intensity		**0.002**		0.462		0.7942		** < 0.001**
	*Low–high*		***0.011***						***0.003***
	*Moderate–high*		*0.916*						***0.018***
	*Moderate–low*		***0.029***						*0.532*
	Drying days		** < 0.001**		0.197		**0.001**		** < 0.001**
	*10–30*		***< 0.001***				***<0.001***		***0.003***
	*10–90*		***< 0.001***				***<0.001***		***0.003***
	*30–90*		***0.016***				*0.796*		***0.027***
**Fungal**									
	Sediment type	**0.001**	0.126	**0.001**	**0.001**	**0.001**	**0.015**	0.100	**<0.001**
	Drying intensity		**0.024**		0.193		0.191		**<0.001**
	*Low–high*		***0.019***						***0.024***
	*Moderate–high*		*0.784*						*0.195*
	*Moderate–low*		*0.087*						*1.000*
	Drying days		**0.004**		**0.001**		**0.005**		**<0.001**
	*10–30*		***0.004***		*0.208*		*0.208*		***0.003***
	*10–90*		*0.728*		***0.001***		***0.001***		***0.003***
	*30–90*		***0.029***		*0.095*		*0.091*		***0.018***

*For the prior-to-drying phase *P*-values are derived from a one-way PERMANOVA comparison between sediment types (non-enriched and enriched), while for the drying phase *P*-values is derived from three-way PERMANOVAs for sediment type, drying intensity, and drying duration comparisons. Adjusted *P*-values for Tukey’s HSD and pairwise tests (italicized text and numbers) indicate significant variability among drying intensities (low, moderate and high) and drying durations (10, 30 and 90 days). Significant *P*-values (*P* < 0.05) are marked in bold.*

### Bacterial and Fungal Diversity

**FIGURE 2:**
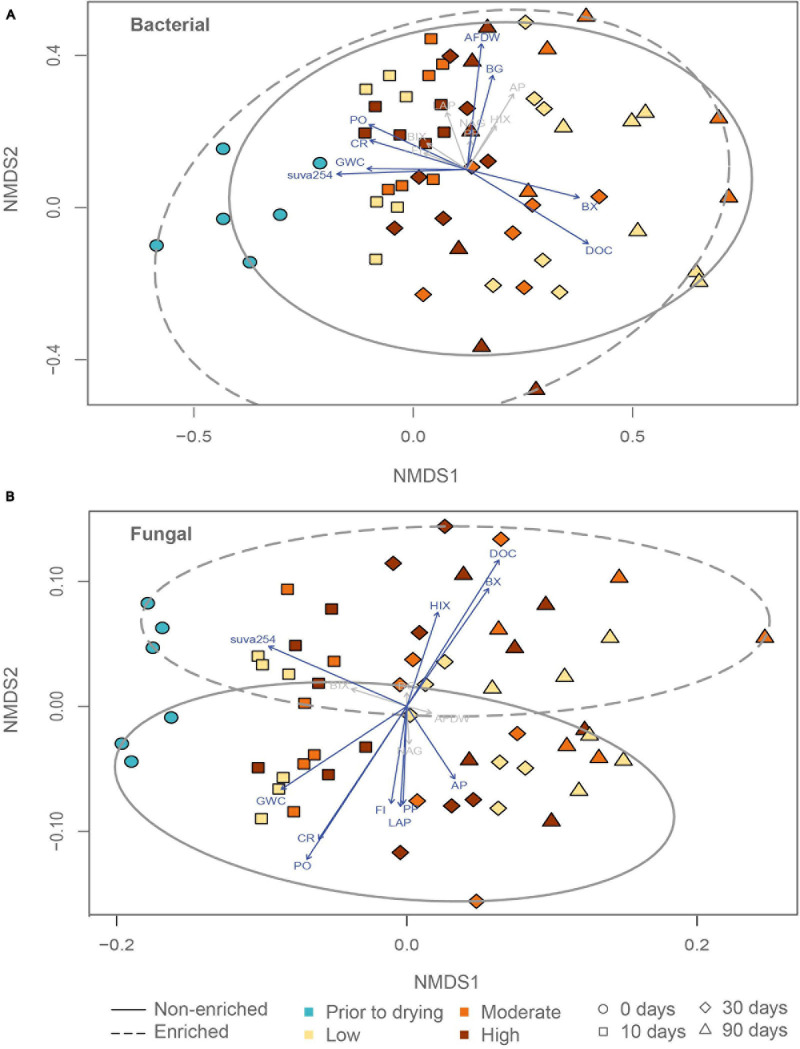
Non-metric multidimensional scaling ordination (nMDS) of **(A)** bacterial and **(B)** fungal communities in relation to a suite of environmental variables linked to organic matter content (ash free dry weight (**AFDW**), dissolve organic carbon (**DOC**), fluorescence index (**FI**), humification index (**HIX**), biological index (**BIX**), and **suva254**), sediment gravimetric water content (**GWC**), and all microbial functions tested (community respiration (**CR**), β-glucosidase (**BG**), β-xylosidase (**BX**), phenol peroxidase (**PP**), phenol oxidase (**PO**), alkaline phosphatase (**AP**), chitinase (**NAG**) and leucine aminopeptidase (**LAP**)).

Before and after drying, bacterial richness and the Shannon diversity index did not differ significantly between enriched and non-enriched sediment. The bacterial Shannon diversity index significantly decreased through drying in non-enriched and enriched sediment (Supplementary Figure 3 and Table 1). Sediment type, drying duration and drying intensity significantly affected the bacterial community assembly (Figure 2 and Table 1). Surprisingly, the bacterial community structure of non-enriched sediment shifted less with increasing drying time in the high-intensity drying treatment than in the other treatments (Figure 2). Some environmental variables linked to sediment organic matter content (DOC, AFDW, suva254), sediment gravimetric water content, and some microbial functions (Community respiration, β-glucosidase, β-xylosidase, phenol oxidase) were found to be significantly correlated with the shifts in the bacterial community (Figure 2 and Supplementary Table 3).

Under flowing conditions, prior to drying, fungal richness and Shannon diversity index was significantly higher in enriched sediments (Supplementary Figure 3 and Table 1). Fungal richness in the enriched sediment treatments decreased significantly from 10 to 90 days of drying, whereas non-enriched sediment values remained more stable during the same period. The fungal community clustered primarily based on sediment type (Figure 2 and Table 1). However, drying duration and intensity significantly affected the fungal community assembly. Most environmental variables linked to organic matter content in the sediment (DOC, suva254, HIX, FI), sediment gravimetric water content, and few microbial functions tested (Community respiration, β-xylosidase, phenol oxidase, and phenol peroxidase) were significantly correlated to shifts in the fungal community structure (Figure 2 and Supplementary Table 3).

### Bacterial Taxa

**FIGURE 3:**
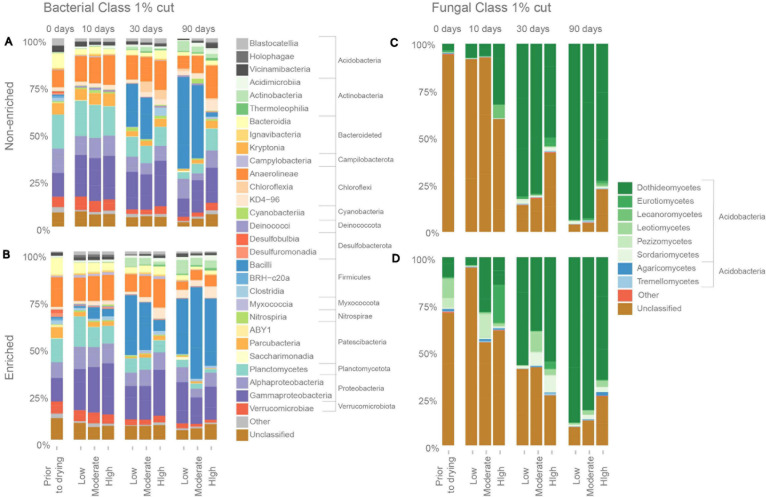
Relative average (*n* = 3) abundance of major bacterial classes in **(A)** non-enriched and **(B)** enriched sediment, and relative abundance of major fungal classes in **(C)** non-enriched and **(D)** enriched sediment under various drying intensities (low, moderate, and high) and after different drying durations (0, 10, 30, and 90 days).

**FIGURE 4:**
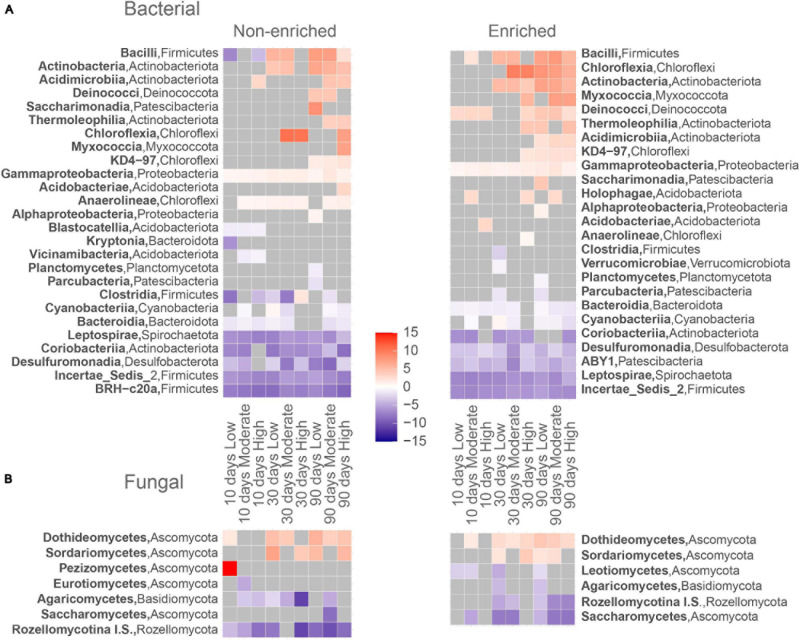
Log2-fold change heatmap for abundant (>1%) significantly responsive **(A)** bacterial and **(B)** fungal ASV’s for each treatment to its corresponding prior to drying assembly. Assigned red squares represent ASV’s that significantly increase (tolerant to drying) and blue squares ASV’s that significantly decrease (sensitive to drying).

Prior to drying, under flowing conditions, non-enriched sediments contained 37 bacterial classes from 16 phyla and enriched sediments contained 56 classes from 19 phyla (Figure 3). The dominant bacterial classes in both sediment types were Planctomycetes (Planctomycetota; 18.1 ± 0.2% and 12.5 ± 2.3% of the bacterial read abundance for non-enriched and enriched sediment, respectively), Alphaproteobacteria (Proteobacteria; 12.9 ± 1.9% and 8.3 ± 4.1%), Anaeroline (Chloroflexi, 9.14 and 3.3% and 15.8 ± 3.7%) and Gammaproteobacteria (Proteobacteria, 12.7 ± 1.5% and 12.5 ± 1.8%). At the end of the experiment (90 days), Bacilli was the dominant bacterial class in all treatments, except for high-intensity drying in non-enriched sediment. The relative abundance of Bacilli increased drastically after 90 days of drying, with values ranging from 29.3% to 48.6% of the total bacterial read abundance in all treatments except for high-intensity drying in non-enriched sediment, where the Bacilli relative abundance was only 2.3%. Other dominating bacterial classes after 90 days of drying (all treatment and sediment types combined) included Gammaproteobacteria (Proteobacteria), Alphaproteobacteria (Proteobacteria) and Anaerolineae (Chloroflexi). Bacilli (Firmicutes), Actinobacteria (Actinobacteriota), Thermoleophilia (Actinobacteriota) and Acidimicrobiia (Actinobacteriota) ASVs were among the most drying-tolerant bacterial classes across drying intensities treatments and both sediment types, meaning that their relative abundance increased significantly with drying (Figure 4). In contrast, the bacterial classes most sensitive to drying (i.e. their relative abundance decreased significantly) included BRH-c20a (Firmicutes), Incertae Sedis 2 (Firmicutes) and Desulfuromonadia (Desulfubacterota) for non-enriched sediment and Incertae ABY1 (Patescibacteria), Leptospirae (Spirochaetota), and BRH-c20a (Firmicutes) in enriched sediment.

### Fungal Taxa

Under flowing conditions, prior to drying, we identified 11 fungal classes from 5 phyla in non-enriched sediments and 16 classes from 6 phyla in enriched ones (Figure 3). The most dominant fungal classes in both sediment types were Dothideomycetes (Ascomycota; 3.6% ± 2.9% and 10.6% ± 2.2% of fungal read abundance for non-enriched and enriched sediment, respectively), Leotiomycetes (Ascomycota; 0.4% ± 0.3% and 10.5% ± 9.3%) and Pezizomycetes (Ascomycota; 0.1% ± 0.1% and 5.6% ± 8.6%). After 90 days of drying, Dothideomycetes was by far the most dominant fungal class in all of our treatments, making up 65.1% to 94.0% of the fungal reads. Nevertheless, unclassified fungal ASVs accounted for 94.1% of the fungal ASVs in non-enriched and 70.3% in enriched sediment prior to drying, but only for 13.3% after 90 days of drying. Dothideomycetes (Ascomycota) and Sordariomycetes (Ascomycota) fungal ASVs were found to be tolerant to drying in most treatments (Figure 4). In contrast, Rozellomycotina I.S. (Rozellomycota) and Saccharomycetes (Ascomycota) classes were observed to be sensitive to drying (Figure 4).

### Initial Community Respiration and Drying Sediment CO_2_ Fluxes

**FIGURE 5:**
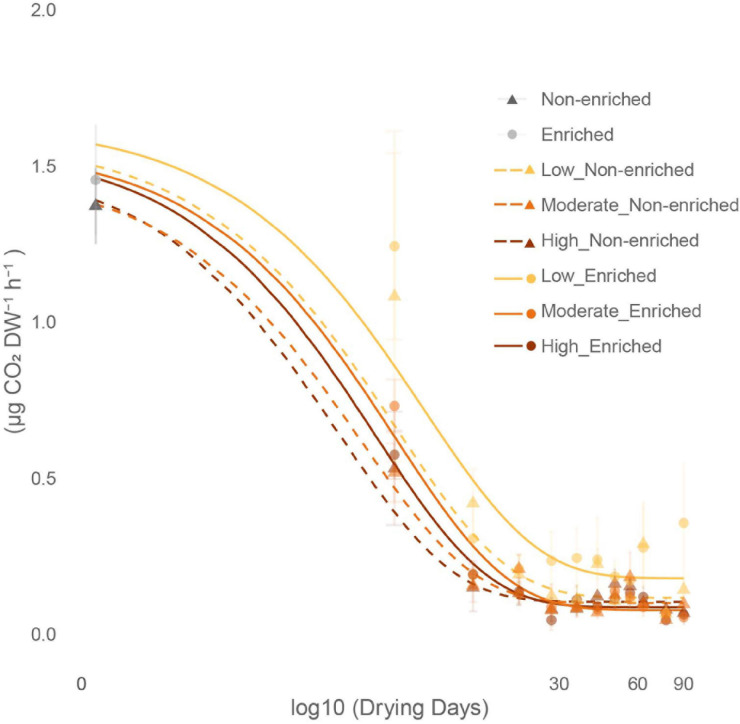
Effect of sediment type (non-enriched or enriched) and drying intensity (low, moderate, or high) on the average (mean + SE, *n* = 3) CO_2_ production decline throughout the drying experiment (0 to 90 days).

Under flowing conditions, prior to drying, community respiration converted to CO_2_ production was significantly greater in enriched (4.55 ± 0.51 μg CO_2_ DW^–1^ h^–1^) than in non-enriched sediment (2.86 ± 0.51 μg CO_2_ DW^–1^ h^–1^; Supplementary Table 4). Initial drainage of pore water from the sediment at day 0 caused a clear drop in the microbial community activity in both sediment types registered as CO_2_ fluxes. However, a non-significant trend remained of higher activity in the enriched sediment (1.45 ± 0.52 CO_2_ DW^–1^ h^–1^) than in the non-enriched sediment (1.37 ± 0.37 μg CO_2_ DW^–1^ h^–1^). During the early drying phase, between 0 and 30 days, CO_2_ fluxes decreased exponentially in all treatments and stabilized after 30 days at an average rate of 0.12 ± 0.10 μg CO_2_ DW^–1^ h^–1^ for both sediment types and all three drying treatments (Figure 5).

### Extracellular Enzymatic Activity

**FIGURE 6:**
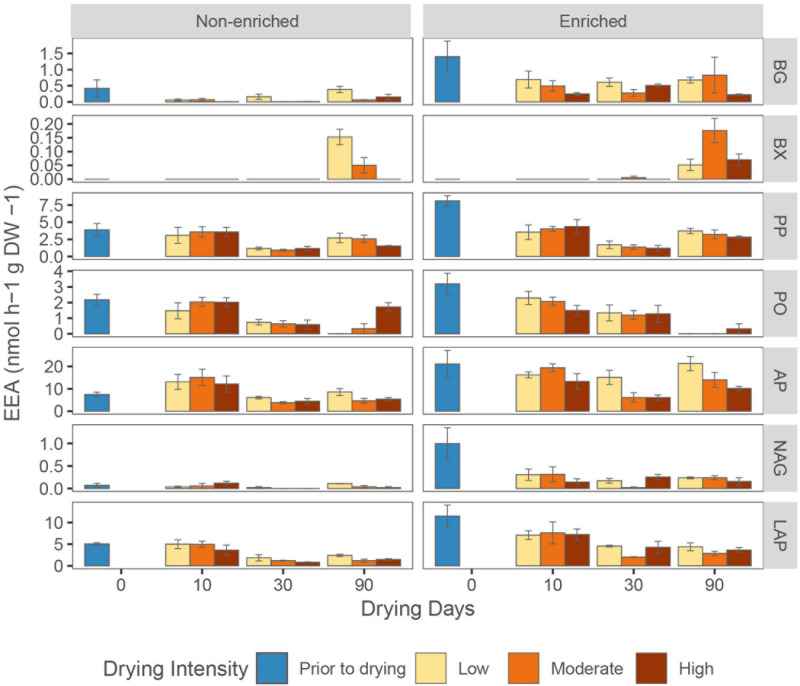
Average (mean + SE, *n* = 3) sediments extracellular enzymatic activities (EEA) for β-glucosidase (**BG**), β-xylosidase (**BX**), phenol peroxidase **(PP)**, phenol oxidase (**PO**), alkaline phosphatase (**AP**), chitinase (**NAG**), and leucine aminopeptidase (**LAP**) under various drying intensities (low, moderate, and high) at different drying days (0, 10, 30, and 90).

β-glucosidase, alkaline phosphatase, chitinase, leucine aminopeptidase, and phenol peroxidase activities were significantly higher in enriched sediment, whereas β-xylosidase and phenol oxidase activities were not affected by sediment type (Figure 6 and Supplementary Table 4). Over the whole drying period, the activity of β-glucosidase, β-xylosidase and alkaline phosphatase was lowest in the high-intensity drying treatment (Supplementary Table 4). However, drying duration influenced enzyme activities in different ways. Alkaline phosphatase and phenol peroxidase activity significantly decreased from 10 to 30 days of drying, but increased again between 30 to 90 days (Figure 6). Phenol oxidase activity decreased gradually during drying in all treatments except for high-intensity drying in non-enriched sediment, where it decreased from 10 to 30 days but rose again from 30 to 90 days (Figure 6). Moreover, while leucine aminopeptidase activity steadily decreased with drying time, β-xylosidase activity greatly increased after 90 days of drying in all treatments except for high-intensity drying in the non-enriched sediment (Figure 6).

### DOM Quality and DOC Quantity

The DOM leachate level of humification based on HIX values showed a significant increase between 10 and 30 days of drying and then decreased significantly between 30 and 90 days, regardless of drying intensity or sediment type (Supplementary Figure 4 and Supplementary Table 2). SUVA254, a proxy for aromatic DOM content, tended to be higher in non-enriched sediment (1.18 ± 0.13) than in enriched sediment (0.6 ± 0.03) prior to drying, but the difference was not significant. Similar to HIX dynamics, SUVA254values increased significantly between 10 and 30 days, followed by a significant decrease between 30 and 90 days of drying (Supplementary Figure 4 and Supplementary Table 2). SUVA254 was lower in the low-intensity drying treatment than in the moderate drying treatment. BIX, an indicator for autochthonous microbial-derived DOM content, remained low (<1) throughout the experiment with no significant differences among treatments, suggesting an allochthones DOM origin. According to the FI values, drying intensity generally did not influence the ratio of terrestrial- to microbial-derived DOM. However, we did observe a significant decrease in FI for enriched sediments between 10 and 30 days. DOC quantity varied little within the first 30 days of drying but was considerably and significantly increased at day 90 in all treatments (Supplementary Figure 4 and Supplementary Table 2).

## Discussion

### Microbial Community Structure Response to Drying Treatments

Results from this study demonstrate that drying intensity affects the bacterial and fungal community assembly throughout riverbed drying. In contrast to our first hypothesis, the bacterial, and fungal riverbed community composition shifted more strongly under milder drying intensities than under high drying intensity. Milder drying intensities might have allowed microbial cells to proliferate or, on the contrary, to prepare for drought and adapt to the new conditions in the drying sediment, for example by forming spores. Taxa able to grow or persist under milder drying conditions may have profited from more sensitive taxa by scavenging and taking up nutrients from these recently lysed cells. Similar findings were reported by Gionchetta et al. (2019) in riverbeds, where 5 months of drying attenuated by sporadic storms triggered a stronger effect on the sediment microbial community composition in comparison to dried sediment without storm events. This highlights the importance of precipitation events on the microbial assembly during drying.

After 90 days of drying, Bacilli (Firmicutes) were by far the dominant class in almost all treatments. Firmicutes prevalence and growth are well documented in dry sediments and soils (Fazi et al., 2008; Timoner et al., 2014; Gionchetta et al., 2019). Firmicutes are Gram-positive bacteria known to resist drying and osmotic stress (Fierer et al., 2003; Schimel et al., 2007), which can be partly attributed to their endospore-forming capabilities. For instance, endospore-forming cells from the genus Bacillus were less severely affected by soil desiccation than non-endospore-forming Bacillus cells (Meisner et al., 2015). Other known Bacilli survival strategies to overcome stress, including scavenging and uptake of genetic material and antibiotic production to suppress competition (Errington, 2003), might have even reinforced the dominance of Bacilli in the low- and moderate-intensity drying treatments, contributing to the stronger change in community composition. Nevertheless, Bacilli colonization, growth or endospore formation might have been partly inhibited under the harshest drying treatment in non-enriched sediment, as they represented only a minority of the bacterial community at the end of the experiment. In the non-enriched sediment, drying might have been too strong and rapid for Bacilli to take on strategies such as spore forming. However, this interpretation should be taken with caution, as the DNA extraction method used in this experiment was not specific for spore-forming DNA cells, and spore-forming taxa might therefore have been overlooked.

Dothideomycetes, the most diverse and numerous fungal class among Ascomycetes (Ametrano et al., 2019), largely dominated the fungal community in all treatments after 90 days of drying. Several Dothideomycetes (e.g., black meristematic fungi) are extremotolerant fungi, capable of resisting extreme temperatures, solar radiation and drought stress (Gostinčar et al., 2012), which is in line with their prevalence at the end of our experiment when the sediment was the driest. Similarly, in previous studies an increasing dominance of Dothideomycetes was reported in surface sediment from a Mediterranean stream (Gionchetta et al., 2019). However, especially before drying, unclassified ASVs represented the majority of the fungal assembly in all treatments, due to biases arising from limited fungal taxa identification. The diversity and abundance of DNA barcode database fungi, especially aquatic fungi, are still poorly described compared to prokaryotes (Marie Booth et al., 2019). Hence, the dynamics of fungal taxa during sediment drying are obscured by the lack of aquatic fungal taxa in the reference database. However, the observed decrease in the proportion of unclassified fungal ASVs with increasing drying time enabled the recognition of specific diversity and composition dynamics among treatments toward the end of the experiment.

### Microbial Functions Response to Drying Treatments

Confirming our second hypothesis, drying affected sediment CO_2_ fluxes (a proxy for community respiration) faster than it affected the bacterial and fungal community structure. These results confirmed the conceptual framework predicting a lower resistance of OM and nutrient cycling to drying conditions than the microbial community structure (Arce et al., 2019). Functional plasticity, i.e. the capacity of microbial cells to cope with environmental changes by adapting their performance (Evans and Hofmann, 2012), could be the mechanism behind the faster response to drying observed in the CO_2_ fluxes. In this sense, microbes would have quickly reduced their metabolic activity as a strategy to overcome drying, allowing them to maintain their community abundance, diversity and composition for a few days, but with immediate consequences for CO_2_ production. Further, the parallel decline in sediment moisture and CO_2_ production with increasing drying time confirmed that moisture is the main controlling factor for sediment CO_2_ production (Keller et al., 2020). Similar moisture declines subsequent to drying have been observed in previous studies simulating drying events in Mediterranean (Amalfitano et al., 2008; Timoner et al., 2012) and temperate (Pohlon et al., 2013) rivers.

Despite responding at different times during the drying experiment, the dynamics of CO_2_ fluxes and bacterial abundance were strongly correlated. This is not surprising, as bacteria are the dominant heterotrophic microorganisms in river sediment (Marxsen, 2011) and are responsible for most C mineralization processes (Amalfitano et al., 2008). A similar decline in sediment bacterial abundance during drying periods has been reported in previous studies (Amalfitano et al., 2008; Pohlon et al., 2013; Gionchetta et al., 2018). Contrary to bacteria, fungal abundance did not show any trend throughout the drying period, indicating a greater potential of fungi to cope with sediment drying than bacteria and matching findings reported by Gionchetta et al. (2019).

Drying duration negatively affected the activities of the extracellular enzymes alkaline phosphatase, leucine aminopeptidase, phenol oxidase and phenol peroxidase, and it positively affected β-xylosidase activity. However, the response of extracellular enzymatic activities to drying was delayed in comparison to that of CO_2_ production. One explanation may be the persistence of extracellular enzyme molecules in drying sediments, as is the case in soils (Gómez et al., 2020). For instance, Zoppini and Marxsen, 2011 suggested that hydrolytic enzymes may be preserved during drying even when the cells from which they originated become non-viable. These mechanisms may have made it possible for extracellular enzyme activities to withstand drying events, explaining their mild effects compared with CO_2_ fluxes.

### Sediment OM and Microbial Community Structure and Functions During Drying

Sediment OM quantity and quality impacted the microbial functions and community structure assembly during drying, in line with our third hypothesis. Greater OM content in sediment reduced the effect of drying intensity. For instance, the bacterial composition in sediment exposed to high-intensity drying (no rain and no shade) differed strongly between non-enriched and OM-enriched sediments at the end of the experiment, with OM-enriched sediment having a composition more similar to the milder drying treatments. Because gravimetric water content was similar in the two sediment types, the reduced effect of drying observed in enriched sediment might be due to the greater OM concentration, which may have allowed greater resource availability for the microbial communities. Sediment OM quality and quantity also strongly affected most extracellular enzymatic activities. Sediment EEA values were generally low compared to values reported in similar studies (Freixa et al., 2016; Gionchetta et al., 2018), and this may be partly due to the sufficient availability of DOM throughout our experiment (Jansson et al., 1988; Romaní and Sabater, 1998). Hence, the small HIX and SUVA254 values observed in all treatments throughout our experiment suggest the presence of fresh labile leachate fraction of the DOM (Hansen et al., 2016). This notwithstanding, most extracellular enzymes tested in our experiment (five out of seven) had higher activities in OM-enriched sediments, suggesting a need for nutrient acquisition by the microbial cells. Higher extracellular enzymatic activity in enriched sediment also correlated with higher CO_2_ fluxes and with generally higher bacterial and fungal abundance.

Dissolved organic carbon quantity remained stable in the early drying phase of our experiment (0 to 30 days), but unexpectedly increased at the end of the experiment (90 days). A rise in DOC due to microbial lysis or a release of exudates subsequent to cell damage, as reported previously for drying riverbed sediment (von Schiller et al., 2015), is unlikely in our study, however, as bacterial and fungal abundance substantially declined between 10 and 30 days of drying, while DOC increased only after 90 days. The observed increase may be attributed to drought-induced effects on leached DOC solubility, mainly caused by alterations in pH and ionic strength, as reported in peatland soils (Clark et al., 2012; Ritson et al., 2017). Also, DOC released by decomposition throughout drying may have exceeded DOC uptake for microbial respiration (Pastor et al., 2003), resulting in an increase in DOC in sediments. Nevertheless, further research is needed to better comprehend what factors led to such sediment DOC leachate dynamics during drying.

## Conclusion

We examined riverbed sediment microbial functions and community structure responses under various drying intensities in river sediments that differed in OM content. We found that less severe drying treatments triggered a more rapid and drastic change in microbial community richness and composition, independent of the sediment OM content. CO_2_ fluxes, a proxy for sediment community respiration, were more rapidly and strongly affected by drying than microbial community abundance and diversity. Finally, sediment OM quantity and quality significantly impacted the microbial community structure and functions throughout drying. Our findings demonstrate a strong impact of drying on stream sediment microbial structure and functions, with potentially large consequences on the biogeochemical dynamics of temperate rivers that have recently become non-perennial/intermittent.

## Data Availability Statement

The datasets presented in this study can be found in online repositories. The names of the repository/repositories and accession number(s) can be found below: https://www.ncbi.nlm.nih.gov/, PRJNA703405.

## Author Contributions

JS, MM, CM-L, and AF designed the study. JS performed the experiment and wrote the manuscript with the help of AF, CM-L, and MM. All authors contributed to the article and approved the submitted version.

## Conflict of Interest

The authors declare that the research was conducted in the absence of any commercial or financial relationships that could be construed as a potential conflict of interest.
