# Supplementary material for: Attributes of Drying Define the Structure and Functioning of Microbial Communities in Temperate Riverbed Sediment
Source: Front Microbiol. 2021 Jun 14;12:676615. doi: 10.3389/fmicb.2021.676615 (PMC8236957; doi:10.3389/fmicb.2021.676615)
Supplement: Supplementary file 1 [file Data_Sheet_1.PDF]

Supplementary Material

Supplementary Table S1 | Sediment sieve size distribution, expressed as percentages of the total weight, for non-enriched and enriched sediment prior to drying.

	**Non-enriched (%)**	**Enriched (%)**
**>2 mm**	1.197	0.741
**2–0.63mm**	9.803	10.325
**0.63–0.2 mm**	70.695	70.297
**0.2–0.063 mm**	18.246	18.506
**<0.063 mm**	0.059	0.131

Supplementary Table S2 | P-values from PERMANOVAs and post-hoc tests (Tukey's HSD) for sediment organic matter and environmental variables properties. For the prior-to-drying phase P-values are derived from a one-way PERMANOVA comparison between sediment types (non-enriched and enriched), while for the drying phase P-values is derived from three-way PERMANOVAs for sediment type, drying intensity, and drying duration comparisons. Adjusted P-values for Tukey's HSD tests indicate significant variability among drying intensities (low, moderate and high) and drying durations (10, 30 and 90 days).

	**DOC**		**bix**		**hix**		**fi**		**SUVA254**	
	Prior to drying	Drying	Prior to drying	Drying	Prior to drying	Drying	Prior to drying	Drying	Prior to drying	Drying
Sediment Type	1.000	0.546	1.000	0.699	1.000	0.363	1.000	0.5904	1.000	0.157
Drying Intensity		0.884		0.276		0.732		0.266		**0.007**
low–high										0.057
moderate–high										0.614
moderate–low										**0.005**
Drying Days		**<0.001**		0.684		**<0.001**		**<0.001**		**<0.001**
10–30		0.936				**<0.001**		**0.015**		**<0.001**
10–90		**<0.001**				0.946		**0.005**		0.680
30–90		**<0.001**				**<0.001**		0.914		**<0.001**

	**AFMW**		**GWC**		**Temperature**		**pH**	
	Prior to drying	Drying	Prior to drying	Drying	Prior to drying	Drying	Prior to drying	Drying
Sediment Type	0.100	**<0.001**	n/a	0.1326	n/a		1.000	
Drying Intensity		0.953		**<0.001**		**0.036**		
low–high				**<0.001**		**<0.001**		
moderate–high				0.574		0.145		
moderate–low				**<0.001**		**<0.001**		
Drying Days		**0.027**		**<0.001**				
10–30		0.829		**<0.001**				
10–90		0.134		**<0.001**				
30–90		**0.039**		0.972				

Supplementary Table S3 | Statistics for continuous variables fitted as vectors on **(A)** bacterial and **(B)** fungal ordination plots (nMDS) .

	(A) Bacterial				(B) Fungal			
	**NMDS1**	**NMDS2**	**r2**	**Pr(>r)**	**NMDS1**	**NMDS2**	**r2**	**Pr(>r)**
**β-glucosidase**	0.2282	0.9736	0.1219	**0.0270** *	-0.003	1.000	0.002	0.943
**β-xylosidase**	0.9596	-0.2814	0.1297	**0.0250** *	0.513	0.859	0.218	**0.003** **
**Alkaline phosphatase**	0.0976	0.9952	0.0207	0.5435	0.495	-0.869	0.081	0.099 .
**Leucine aminopeptidase**	0.4644	0.8856	0.0951	0.0519 .	-0.055	-0.998	0.116	0.034 *
**Chitinase**	-0.2921	0.9564	0.0469	0.2348	0.054	-0.999	0.017	0.650
**Phenol oxidase**	-0.8813	0.4725	0.1175	**0.0270** *	-0.491	-0.871	0.357	**0.001** ***
**Phenol peroxidase**	0.1066	0.9943	0.0112	0.6973	-0.032	-0.999	0.110	**0.040** *
**AFDW**	0.0975	0.9952	0.2084	**0.0030** **	0.955	-0.298	0.006	0.840
**DOC**	0.8119	-0.5838	0.2120	**0.0010** ***	0.476	0.879	0.320	**0.001** ***
**BIX**	-0.7943	0.6076	0.0242	0.4905	-0.939	0.345	0.030	0.420
**FI**	-0.9128	0.4085	0.0212	0.5365	-0.139	-0.990	0.111	**0.038** *
**HIX**	0.4938	0.8696	0.0328	0.3836	0.273	0.962	0.111	**0.038** *
**SUVA254**	-0.9991	-0.0417	0.1614	**0.0070** **	-0.893	0.451	0.206	**0.002** **
**GWC**	-1.0000	0.0093	0.0951	**0.0420** *	-0.793	-0.609	0.215	**0.001** ***
**Community respiration**	-0.9431	0.3324	0.1008	**0.0358** *	-0.496	-0.868	0.271	**0.001** ***

Supplementary Table S4 | P-values from PERMANOVAs and post-hoc tests (Tukey's HSD) for sediment microbial functioning properties. For the prior-to-drying phase P-values are derived from a one-way PERMANOVA comparison between sediment types (non-enriched and enriched), while for the drying phase P-values is derived from three-way PERMANOVAs for sediment type, drying intensity, and drying duration comparisons. Adjusted P-values for Tukey's HSD tests indicate significant variability among drying intensities (low, moderate and high) and drying durations (10, 30 and 90 days).

	**Community Respiration**		**β-glucosidase**		**β-xylosidase**		**Phenol oxidase**	
	Prior to drying	Drying	Prior to drying	Drying	Prior to drying	Drying	Prior to drying	Drying
Sediment Type	**0.001**	0.239	**0.001**	**<0.001**	1.000	**0.143**	0.101	0.739
Drying Intensity				**0.025**		**0.012**		0.343
low–high				**0.045**		**0.045**		
moderate–high				**0.013**		**0.013**		
moderate–low				0.868		0.868		
Drying Days		0.551		0.323		**<0.001**		**<0.001**
10–30						0.994		**<0.001**
10–90						**<0.001**		**<0.001**
30–90						**<0.001**		**0.010**

	**Phenol peroxidase**		**Alkaline phosphatase**		**Chitinase**		**Leucine Aminopeptidase**	
	Prior to drying	Drying	Prior to drying	Drying	Prior to drying	Drying	Prior to drying	Drying
Sediment Type	0.001	**0.042**	**0.001**	**<0.001**	0.100	**<0.001**	**0.001**	**<0.001**
Drying Intensity		0.832		**<0.001**		0.522		0.184
low–high				**0.003**				
moderate–high				0.338				
moderate–low				0.098				
Drying Days		**<0.001**		**<0.001**		0.083		**<0.001**
10–30		**<0.001**		**<0.001**				**<0.001**
10–90		**0.032**		**0.010**				**<0.001**
30–90		**<0.001**		**0.024**				0.930

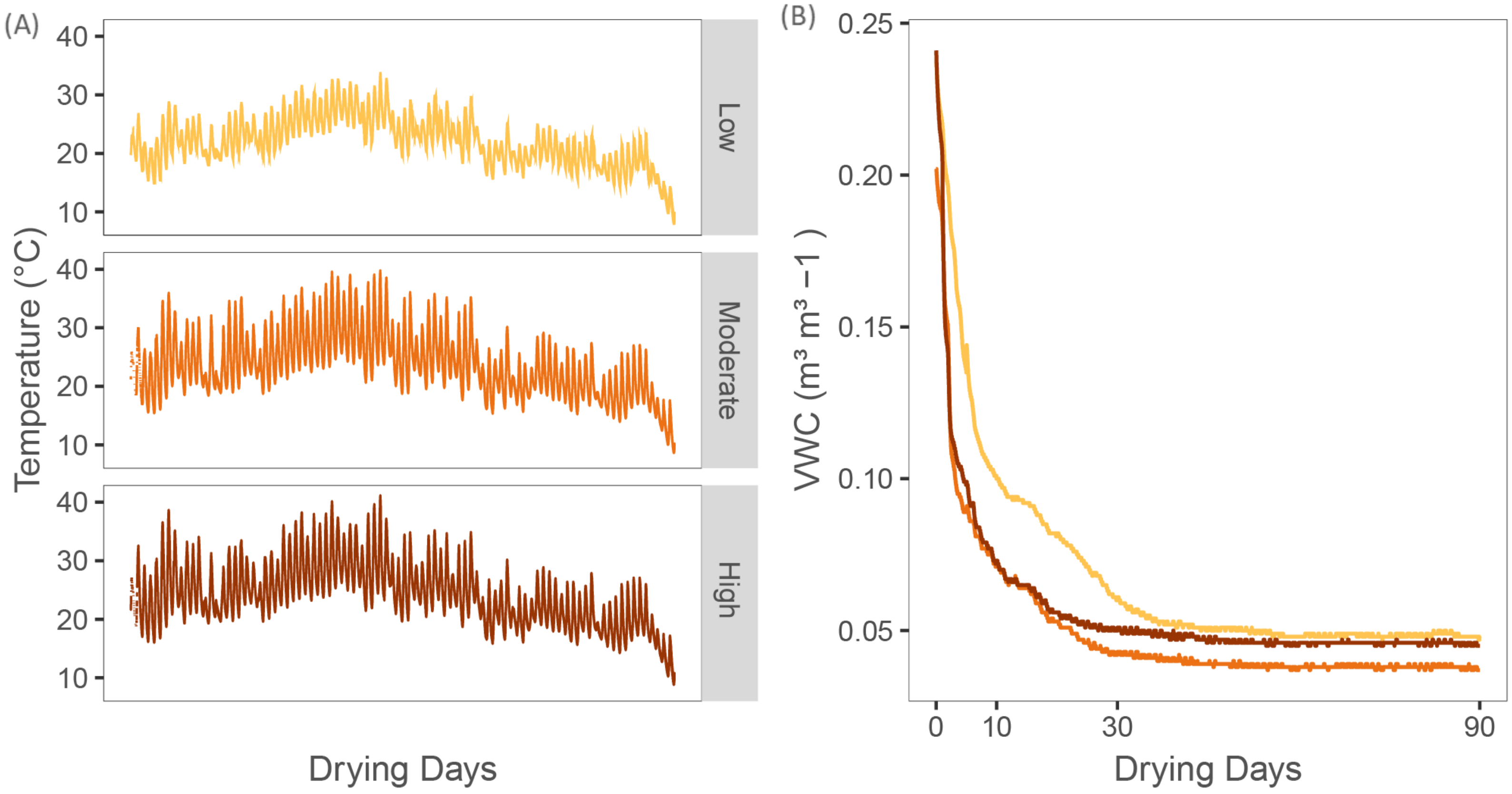

Supplementary Figure S1 | Average **(A)** temperature and **(B)** volumetric water content (VWC) recorded every 30 minutes for non-enriched sediment under the different drying intensities (low, moderate and high).

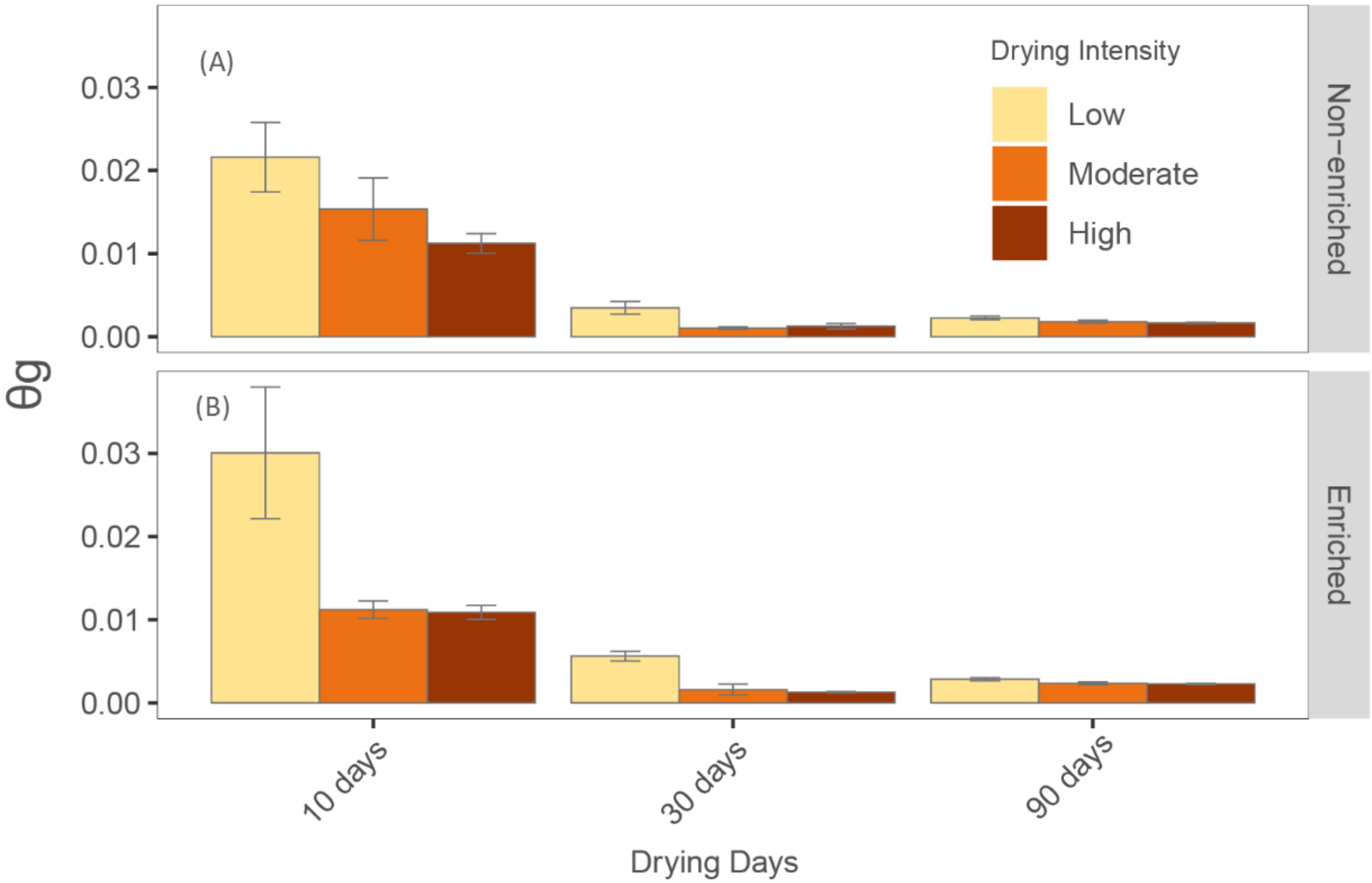

Supplementary Figure S2 | Average (mean ± SE, n = 3) gravimetric water content (ϴg) for **(A)** non-enriched and **(b)** enriched sediment under the different drying intensities (low, moderate and high) and after different drying durations (10, 30 and 90 days).

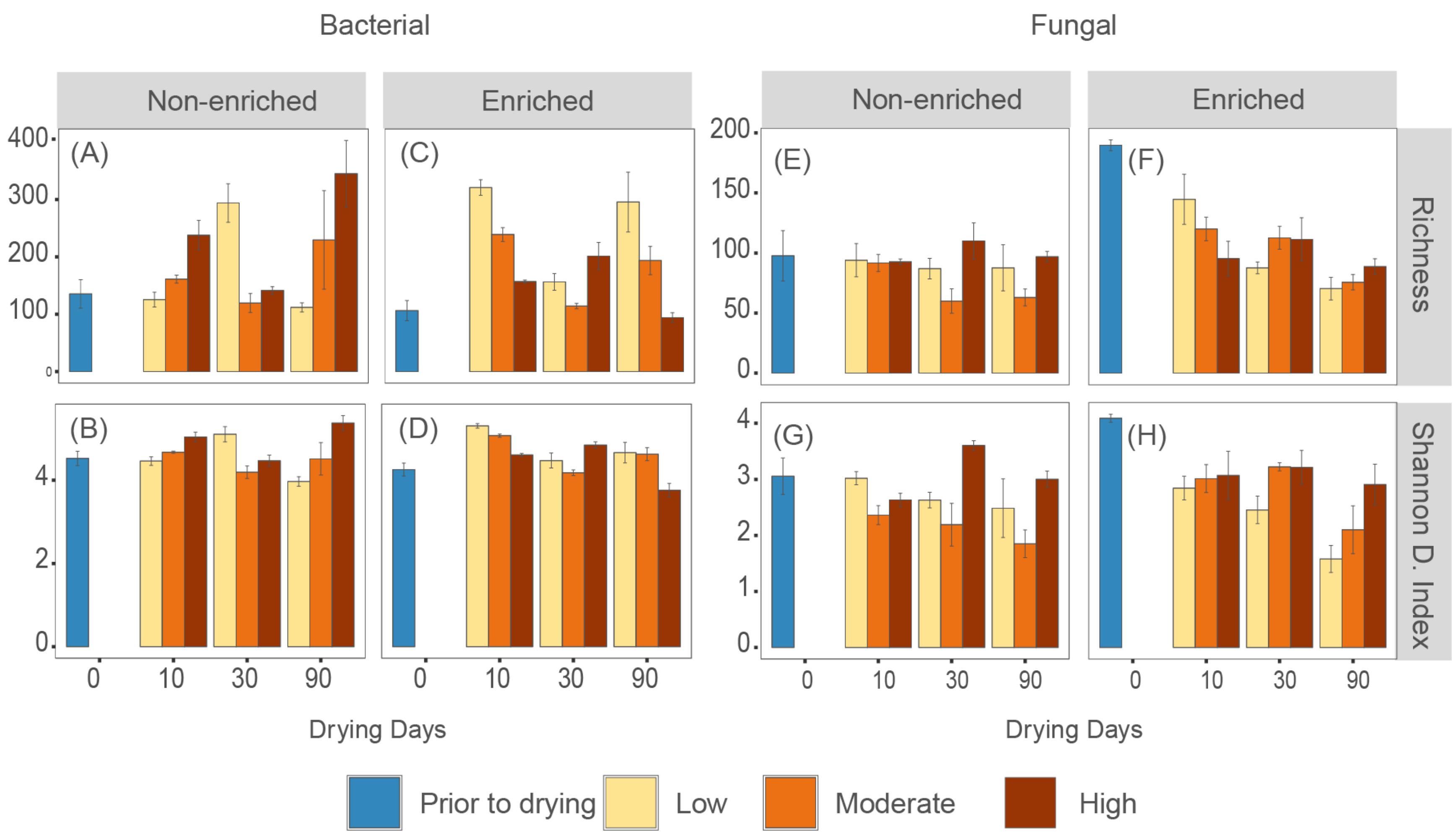

Supplementary Figure S3 | Average (mean ± SE, n = 3) bacterial and fungal richness for **(A, E)** non-enriched and **(B, F)** enriched sediment, and average (mean ± SE, n = 3) bacterial and fungal Shannon diversity index for **(C, G)** non-enriched and **(D, H)** enriched sediment under different drying intensities and after different drying durations.

Sup. Figure 3

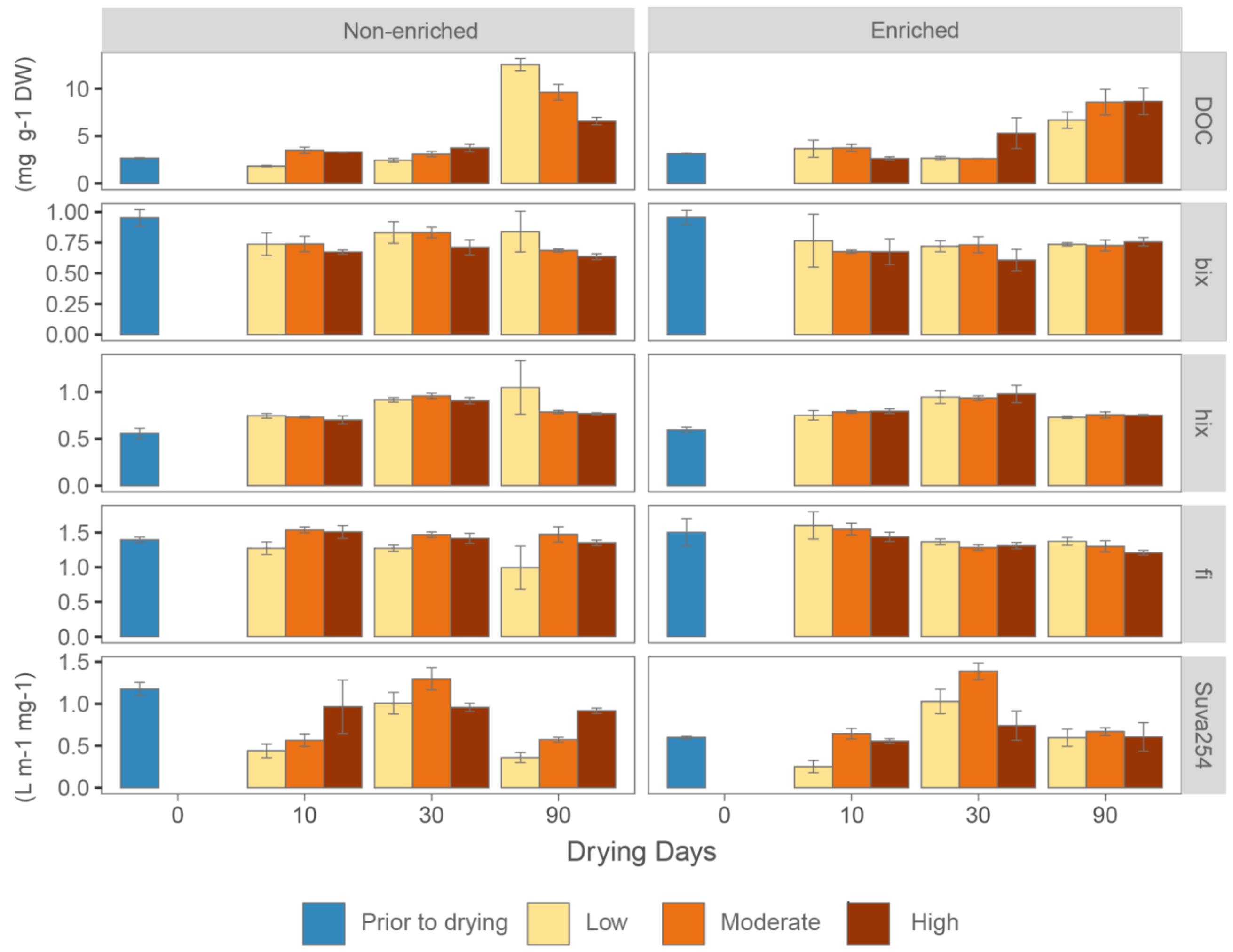

Supplementary Figure S4| Average (mean ± SE, n = 3) DOC quantity and DOM quality parameters (BIX, HIX, FI, SUVA254) under various drying intensities (low, moderate, high) at different drying days (0, 10, 30 and 90).

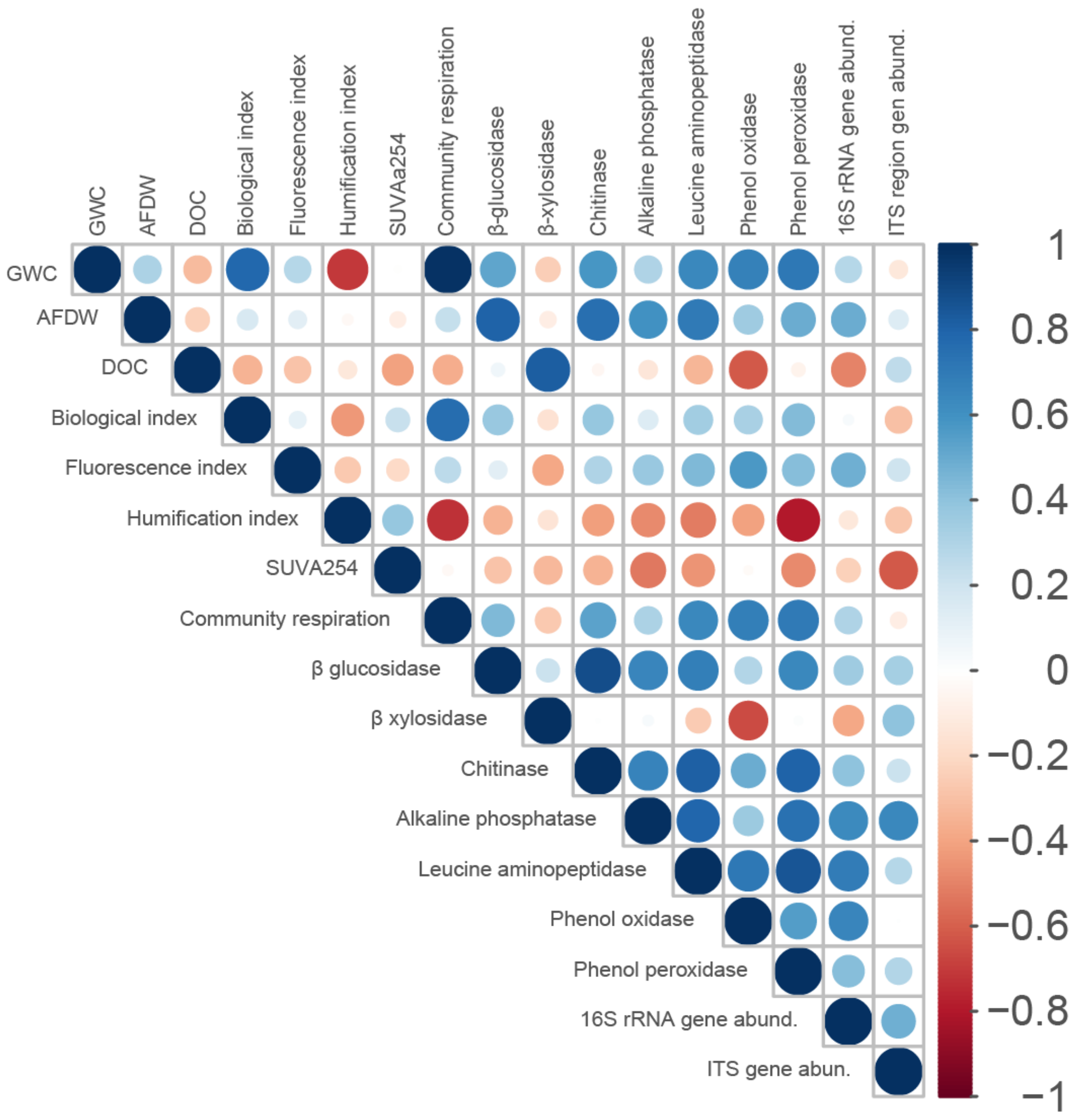

Supplementary Figure S5| Correlation matrix with sediment properties
